# Supplementary material for: The Serbian validation of the Rational-Experiential Inventory-40 and the Rational-Experiential Multimodal Inventory
Source: PLoS One. 2023 Nov 28;18(11):e0294705. doi: 10.1371/journal.pone.0294705 (PMC10684000; doi:10.1371/journal.pone.0294705)
Supplement: S1 Table — (DOCX) [file pone.0294705.s001.docx]

**S1 Table. Standardized loadings for the modified four-factor model for REI-40.**

| **Item** | **Dimension** | **Standardized loading** |
| --- | --- | --- |
| **ra_2r** | Rational Ability | 0.69 |
| **ra_4r** | Rational Ability | 0.55 |
| **ra_8r** | Rational Ability | 0.54 |
| **ra_9r** | Rational Ability | 0.62 |
| **ra_12r** | Rational Ability | 0.37 |
| **ra_13** | Rational Ability | 0.72 |
| **ra_14** | Rational Ability | 0.74 |
| **ra_16** | Rational Ability | 0.30 |
| **ra_17** | Rational Ability | 0.49 |
| **ra_19** | Rational Ability | 0.35 |
| **re_1r** | Rational Engagement | 0.79 |
| **re_3** | Rational Engagement | 0.80 |
| **re_5r** | Rational Engagement | 0.61 |
| **re_6** | Rational Engagement | 0.42 |
| **re_7r** | Rational Engagement | 0.51 |
| **re_10** | Rational Engagement | 0.53 |
| **re_11r** | Rational Engagement | 0.68 |
| **re_15** | Rational Engagement | 0.63 |
| **re_18r** | Rational Engagement | 0.62 |
| **re_20** | Rational Engagement | 0.32 |
| **ea_22r** | Experiential Ability | 0.79 |
| **ea_23** | Experiential Ability | 0.34 |
| **ea_24** | Experiential Ability | 0.35 |
| **ea_27** | Experiential Ability | 0.46 |
| **ea_28** | Experiential Ability | 0.77 |
| **ea_29r** | Experiential Ability | 0.46 |
| **ea_35** | Experiential Ability | 0.82 |
| **ea_37r** | Experiential Ability | 0.85 |
| **ea_39** | Experiential Ability | 0.40 |
| **ea_40r** | Experiential Ability | 0.70 |
| **ee_21** | Experiential Engagement | 0.81 |
| **ee_25** | Experiential Engagement | 0.76 |
| **ee_26** | Experiential Engagement | 0.69 |
| **ee_30r** | Experiential Engagement | 0.62 |
| **ee_31** | Experiential Engagement | 0.44 |
| **ee_32r** | Experiential Engagement | 0.80 |
| **ee_33r** | Experiential Engagement | 0.46 |
| **ee_34r** | Experiential Engagement | 0.56 |
| **ee_36r** | Experiential Engagement | 0.29 |
| **ee_38** | Experiential Engagement | 0.47 |

Note: p < .001 for all loadings
