# Supplementary material for: The Serbian validation of the Rational-Experiential Inventory-40 and the Rational-Experiential Multimodal Inventory
Source: PLoS One. 2023 Nov 28;18(11):e0294705. doi: 10.1371/journal.pone.0294705 (PMC10684000; doi:10.1371/journal.pone.0294705)
Supplement: S2 Table — (DOCX) [file pone.0294705.s002.docx]

**S2 Table. Factor correlations for the modified four-factor model for REI-40.**

| **Factor** | **Rational Ability** | **Rational Engagement** | **Experiential Ability** |  |
| --- | --- | --- | --- | --- |
| **Rational Engagement** | .82 |  |  | |
| **Experiential Ability** | .13 | .12 |  | |
| **Experiential Engagement** | -.05 | .10 | .87 | |

Note: p < .001 for all correlations
