# Supplementary material for: The Serbian validation of the Rational-Experiential Inventory-40 and the Rational-Experiential Multimodal Inventory
Source: PLoS One. 2023 Nov 28;18(11):e0294705. doi: 10.1371/journal.pone.0294705 (PMC10684000; doi:10.1371/journal.pone.0294705)
Supplement: S3 Table — (DOCX) [file pone.0294705.s003.docx]

**S3 Table. Correlations between REI-40 thinking styles and HEXACO personality traits.**

| **Scale** | **1. Rationality** | **2** | **3** | **4** | **5** | **6** | **7** | **8** | **9** | **10** | **11** |
| --- | --- | --- | --- | --- | --- | --- | --- | --- | --- | --- | --- |
| **REI-40** |  |  |  |  |  |  |  |  |  |  |  |
| 1. **Rational Ability (normalized)** | .89** |  |  |  |  |  |  |  |  |  |  |
| 1. **Rational Engagement** | .92** | .64** |  |  |  |  |  |  |  |  |  |
| 1. **Experientiality** | .05 | .02 | .08* |  |  |  |  |  |  |  |  |
| 1. **Experiential Ability** | .09** | .10** | .08* | .91** |  |  |  |  |  |  |  |
| 1. **Experiential Engagement (normalized)** | .02 | -.06 | .08* | .90** | .65** |  |  |  |  |  |  |
| **HEXACO** |  |  |  |  |  |  |  |  |  |  |  |
| 1. **Honesty/Humility** | .09* | .01 | .14** | -.06 | -.07 | -.04 |  |  |  |  |  |
| 1. **Emotionality** | -.13** | -.14** | -.10** | -.06 | -.07 | -.04 | .11** |  |  |  |  |
| 1. **eXtraversion (normalized)** | .25** | .25** | .22** | .20** | .18** | .19** | -.09* | -.12** |  |  |  |
| 1. **Agreeableness** | .04 | -.00 | .07 | -.04 | -.10** | .03 | .27** | -.07 | .04 |  |  |
| 1. **Conscientiousness** | .31** | .32** | .25** | -.11** | -.03 | -.17** | .20** | .15** | .15** | .11** |  |
| 1. **Openness (normalized)** | .47** | .30** | .53** | .13** | .13** | .12** | .08* | .02 | 0.07 | 0.07 | .10** |

* p < .05. ** p < .01.
