# Supplementary material for: The Serbian validation of the Rational-Experiential Inventory-40 and the Rational-Experiential Multimodal Inventory
Source: PLoS One. 2023 Nov 28;18(11):e0294705. doi: 10.1371/journal.pone.0294705 (PMC10684000; doi:10.1371/journal.pone.0294705)
Supplement: S4 Table — (DOCX) [file pone.0294705.s004.docx]

**S4 Table. Model fit for one-factor confirmatory factor models for REIm subscales.**

| **Model** | **χ2(df)** | **χ2/df** | **CFI** | **RMSEA [95% CI]** |
| --- | --- | --- | --- | --- |
| **Rationality 1F** | 383.02 (54) | 7.09 | .923 | .142 [.129 - .155] |
| **Rationality 1F modified** | 200.16 (51) | 3.92 | .965 | .098 [.084 - .113] |
| **Imagination 1F** | 69.46 (35) | 1.98 | .977 | .057 [.037 - -077] |
| **Emotionality 1F** | 173.07 (35) | 4.94 | .900 | .114 [.097 - .131] |
| **Emotionality 1F modified** | 117.10 (34) | 3.44 | .940 | .090 [.072 - .108] |
| **Intuition 1F** | 114.98 (35) | 3.29 | .923 | .087 [.070 - .105] |

Note: The modified rationality model includes three error covariances (for item pairs 8-12, 4-5 and 2-3). The modified Emotionality model includes one error covariance (for the item pair 23-24).
