# Supplementary material for: The Serbian validation of the Rational-Experiential Inventory-40 and the Rational-Experiential Multimodal Inventory
Source: PLoS One. 2023 Nov 28;18(11):e0294705. doi: 10.1371/journal.pone.0294705 (PMC10684000; doi:10.1371/journal.pone.0294705)
Supplement: S5 Table — (DOCX) [file pone.0294705.s005.docx]

**S5 Table. Standardized loadings for the modified one-factor model for REIm Rationality.**

| **Item** | **Dimension** | **Standardized loading** |
| --- | --- | --- |
| **REIM_1r** | Rationality | 0.65 |
| **REIM_2r** | Rationality | 0.40 |
| **REIM_3r** | Rationality | 0.45 |
| **REIM_4** | Rationality | 0.71 |
| **REIM_5** | Rationality | 0.65 |
| **REIM_6** | Rationality | 0.59 |
| **REIM_7** | Rationality | 0.70 |
| **REIM_8r** | Rationality | 0.44 |
| **REIM_9** | Rationality | 0.56 |
| **REIM_10** | Rationality | 0.70 |
| **REIM_11r** | Rationality | 0.25 |
| **REIM_12r** | Rationality | 0.48 |

Note: p < .001 for all loadings
