# Supplementary material for: The Serbian validation of the Rational-Experiential Inventory-40 and the Rational-Experiential Multimodal Inventory
Source: PLoS One. 2023 Nov 28;18(11):e0294705. doi: 10.1371/journal.pone.0294705 (PMC10684000; doi:10.1371/journal.pone.0294705)
Supplement: S6 Table — (DOCX) [file pone.0294705.s006.docx]

**S6 Table. Standardized loadings for the one-factor model for REIm Imagination.**

| **Item** | **Dimension** | **Standardized loading** |
| --- | --- | --- |
| **REIM_13** | Imagination | 0.65 |
| **REIM_14** | Imagination | 0.66 |
| **REIM_15** | Imagination | 0.60 |
| **REIM_16** | Imagination | 0.48 |
| **REIM_17** | Imagination | 0.63 |
| **REIM_18** | Imagination | 0.56 |
| **REIM_19** | Imagination | 0.16 |
| **REIM_20** | Imagination | 0.50 |
| **REIM_21** | Imagination | 0.59 |
| **REIM_22r** | Imagination | 0.49 |
| **REIM_23r** | Imagination | 0.65 |

Note: p < .001 for all loadings
