# Supplementary material for: The Serbian validation of the Rational-Experiential Inventory-40 and the Rational-Experiential Multimodal Inventory
Source: PLoS One. 2023 Nov 28;18(11):e0294705. doi: 10.1371/journal.pone.0294705 (PMC10684000; doi:10.1371/journal.pone.0294705)
Supplement: S7 Table — (DOCX) [file pone.0294705.s007.docx]

**S7 Table. Standardized loadings for the modified one-factor model for REIm Emotionality.**

| **Item** | **Dimension** | **Standardized loading** |
| --- | --- | --- |
| **REIM_24r** | Emotionality | 0.48 |
| **REIM_25** | Emotionality | 0.57 |
| **REIM_26** | Emotionality | 0.64 |
| **REIM_27** | Emotionality | 0.64 |
| **REIM_28** | Emotionality | 0.58 |
| **REIM_29** | Emotionality | 0.68 |
| **REIM_30r** | Emotionality | 0.24 |
| **REIM_31** | Emotionality | 0.36 |
| **REIM_32r** | Emotionality | 0.21 |

Note: p < .001 for all loadings
