# Supplementary material for: The Serbian validation of the Rational-Experiential Inventory-40 and the Rational-Experiential Multimodal Inventory
Source: PLoS One. 2023 Nov 28;18(11):e0294705. doi: 10.1371/journal.pone.0294705 (PMC10684000; doi:10.1371/journal.pone.0294705)
Supplement: S8 Table — (DOCX) [file pone.0294705.s008.docx]

**S8 Table. Standardized loadings for the one-factor model for REIm Intuition.**

| **Item** | **Dimension** | **Standardized loading** |
| --- | --- | --- |
| **REIM_33** | Intuition | 0.41 |
| **REIM_34** | Intuition | 0.82 |
| **REIM_35** | Intuition | 0.78 |
| **REIM_36r** | Intuition | 0.53 |
| **REIM_37r** | Intuition | 0.25 |
| **REIM_38** | Intuition | 0.37 |
| **REIM_39** | Intuition | -0.04 |
| **REIM_40** | Intuition | 0.40 |
| **REIM_41** | Intuition | 0.29 |
| **REIM_42r** | Intuition | 0.30 |

Note: p < .001 for all loadings
