# Supplementary material for: The Serbian validation of the Rational-Experiential Inventory-40 and the Rational-Experiential Multimodal Inventory
Source: PLoS One. 2023 Nov 28;18(11):e0294705. doi: 10.1371/journal.pone.0294705 (PMC10684000; doi:10.1371/journal.pone.0294705)
Supplement: S9 Table — (DOCX) [file pone.0294705.s009.docx]

**S9 Table. Standardized loadings for the modified four-factor model for REIm-13.**

| **Item** | **Dimension** | **Factor 1** |
| --- | --- | --- |
| **REIM_1r** | Rationality | 0.56 |
| **REIM_3r** | Rationality | 0.57 |
| **REIM_7** | Rationality | 0.67 |
| **REIM_9** | Rationality | 0.55 |
| **REIM_13** | Imagination | 0.70 |
| **REIM_14** | Imagination | 0.61 |
| **REIM_15** | Imagination | 0.58 |
| **REIM_24r** | Emotionality | 0.71 |
| **REIM_25** | Emotionality | 0.68 |
| **REIM_26** | Emotionality | 0.50 |
| **REIM_33** | Intuition | 0.31 |
| **REIM_35** | Intuition | 1.00 |
| **REIM_36r** | Intuition | 0.47 |

Note: p < .001 for all loadings
