# Supplementary material for: The Serbian validation of the Rational-Experiential Inventory-40 and the Rational-Experiential Multimodal Inventory
Source: PLoS One. 2023 Nov 28;18(11):e0294705. doi: 10.1371/journal.pone.0294705 (PMC10684000; doi:10.1371/journal.pone.0294705)
Supplement: S10 Table — (DOCX) [file pone.0294705.s010.docx]

**S10 Table. Factor correlations for the modified four-factor model for REIm-13.**

| **Factor** | **Rationality** | **Imagination** | **Emotionality** |
| --- | --- | --- | --- |
| **Imagination** | .45 |  |  |
| **Emotionality** | -.17 | .35 |  |
| **Intuition** | -.08 | .10 | .31 |

Note: p < .001 for all correlations
