# Supplementary material for: The Serbian validation of the Rational-Experiential Inventory-40 and the Rational-Experiential Multimodal Inventory
Source: PLoS One. 2023 Nov 28;18(11):e0294705. doi: 10.1371/journal.pone.0294705 (PMC10684000; doi:10.1371/journal.pone.0294705)
Supplement: S11 Table — (DOCX) [file pone.0294705.s011.docx]

**S11 Table. Correlations between REIm(-13) thinking styles, HEXACO and Disintegration personality traits.**

|  | **1. R** | **2** | **3** | **4** | **5** | **6** | **7** | **8** | **9** | **10** | **11** | **12** | **13** | **14** | **15** | **16** |
| --- | --- | --- | --- | --- | --- | --- | --- | --- | --- | --- | --- | --- | --- | --- | --- | --- |
| **REIm** |  |  |  |  |  |  |  |  |  |  |  |  |  |  |  |  |
| **2. Exp** | -.01 |  |  |  |  |  |  |  |  |  |  |  |  |  |  |  |
| **3. In** | -.13* | .69** |  |  |  |  |  |  |  |  |  |  |  |  |  |  |
| **4. Em** | -.16** | .72** | .32** |  |  |  |  |  |  |  |  |  |  |  |  |  |
| **5. Im-n** | .21** | .73** | .26** | .25** |  |  |  |  |  |  |  |  |  |  |  |  |
| **REIm-13** |  |  |  |  |  |  |  |  |  |  |  |  |  |  |  |  |
| **6. R-n** | .87** | .08 | -.07 | -.11 | .28** |  |  |  |  |  |  |  |  |  |  |  |
| **7. Exp** | -.03 | .84** | .65** | .60** | .57** | .05 |  |  |  |  |  |  |  |  |  |  |
| **8. In-n** | -.07 | .36** | .73** | .12* | .02 | -.04 | .56** |  |  |  |  |  |  |  |  |  |
| **9. Em-n** | -.20** | .62** | .32** | .81** | .23** | -.12* | .68** | .12* |  |  |  |  |  |  |  |  |
| **10. Im-n** | .20** | .61** | .24** | .19** | .81** | .24** | .66** | .04 | .17** |  |  |  |  |  |  |  |
| **HEXACO** |  |  |  |  |  |  |  |  |  |  |  |  |  |  |  |  |
| **11.H** | .06 | .09 | .06 | .01 | .11 | .08 | .11 | -.02 | .10 | .10 |  |  |  |  |  |  |
| **12. E** | -.24** | .33** | .14* | .53** | .06 | -.20** | .31** | -.03 | .55** | .07 | .15* |  |  |  |  |  |
| **13. X-n** | .24** | .08 | .19** | -.08 | 0.05 | .15* | .04 | .09 | -.10 | .08 | .00 | -.02 |  |  |  |  |
| **14. A** | -.09 | -.01 | .06 | -.18** | .10 | -.05 | -.02 | -.09 | -.02 | .07 | .30** | -.09 | .10 |  |  |  |
| **15. C** | .32** | .00 | -.11 | -.02 | .10 | .34** | .04 | -.08 | .02 | .14* | .26** | .12* | .25** | 0.05 |  |  |
| **16. O** | .36** | .50** | .18** | .16** | .68** | .36** | .37** | .02 | .13* | .51** | .05 | -.04 | .16** | .14* | .10 |  |
| **17. D** | -.25** | .07 | .08 | .15** | -.07 | -.20** | .09 | .12* | .12* | -.05 | -.24** | -.06 | -.30** | -.21** | -.30** | -.09 |

* p < .05. ** p < .01.

Note. R – Rational thinking style (normalized in REIm-13); Exp – Experiential thinking style; In – Intuition (normalized); Em – Emotionality (normalized); Im – Imagination (normalized); H – Honesty/Humility; E – Emotionality; X – eXtraversion (normalized); A – Agreeableness; C – Conscientiousness; O – Openness to Experience; D – Disintegration.
