# Supplementary material for: The Serbian validation of the Rational-Experiential Inventory-40 and the Rational-Experiential Multimodal Inventory
Source: PLoS One. 2023 Nov 28;18(11):e0294705. doi: 10.1371/journal.pone.0294705 (PMC10684000; doi:10.1371/journal.pone.0294705)
Supplement: S12 Table — (DOCX) [file pone.0294705.s012.docx]

**S12 Table. Regression analyses with REIm(-13) dimensions as criteria and HEXACO personality traits as predictors.**

| **Criterion** | **F(df)** | **p** | **R^2^** | **Significant predictors^a^** |
| --- | --- | --- | --- | --- |
| **REIm** |  |  |  |  |
| **Rationality** | 24.51(6,295) | <.001 | .33 | E- X+ A- C+ O+ |
| **Experientiality** | 30.60(6,295) | <.001 | .38 | E+ C- O+ |
| **Intuition** | 6.96(6,295) | <.001 | .12 | E+ X+ C- O+ |
| **Emotionality** | 26.87(6,295) | <.001 | .35 | E+ A- O+ |
| **Imagination** | 43.52(6,295) | <.001 | .47 | O+ |
| **REIm-13** |  |  |  |  |
| **Rationality** | 20.64(6,295) | <.001 | .31 | E- A- C+ O+ |
| **Experientiality** | 16.13(6,295) | <.001 | .25 | E+ O+ |
| **Intuition** | 1.57(6,295) | .16 | .03 |  |
| **Emotionality** | 25.03(6,295) | <.001 | .34 | E+ X- O+ |
| **Imagination** | 18.45(6,295) | <.001 | .27 | O+ |

^a^  Significant at p ≤ .005. Predictors significant at .005 < p < .05 are given in grey font. H - Honesty/Humility, E - Emotionality, X - eXtraversion, A - Agreeableness, C - Conscientiousness, O - Openness to experience
