# Supplementary material for: The Serbian validation of the Rational-Experiential Inventory-40 and the Rational-Experiential Multimodal Inventory
Source: PLoS One. 2023 Nov 28;18(11):e0294705. doi: 10.1371/journal.pone.0294705 (PMC10684000; doi:10.1371/journal.pone.0294705)
Supplement: Attachment — Submitted filename: PLOS-One Review.pdf [file pone.0294705.s013.pdf]

Thank you very much for submitting the paper entitled: “The Serbian validation of three versions of the Rational-Experiential Inventory: REI-40, REIm, and REIm-13.” The paper addresses an important topic by examining the validity of a new version of Rational-Experiential scale in two Serbian student samples. However, the paper has some major issues that need to be addressed in the next version. Below are some comments for improving the paper:

1. The introduction is very short and does not properly present the background and address the criticality of the issue and the purpose for conducting this study. Please provide a more detailed introduction to set the stage for the rest of the paper.
2. Please follow the guidelines for the structure of the paper. The sub-sections of Theoretical foundations of rationality and experientiality, Rational-Experiential Inventory: The three versions, current study, etc do not match the guidelines for the paper structure as provided in PLUS-One journal. In addition, they are not well-structured. The literature review section should either be discussed as part of the introduction section or as a separate section under the literature review section with a few introductory paragraphs that explain the structure of the literature review section.
3. More references are required in different parts of the paper. For example, in the first paragraph of “Theoretical foundations of rationality and experientiality” section, no references are mentioned. In addition, a more general explanation of dual process theories and CEST as one of these theories are required in this paragraph. In addition, please use recent references as much as possible throughout the paper.
4. What do you mean by other dual process theories and what are the assumptions for similarities and dissimilarities between other dual process theories and CEST. As far as I know, all dual process theories distinguish between rational and intuitive thinking styles. I think, it is better to mainly concentrate on the theory that is the focus of this paper or rewrite this section to decrease the ambiguities in introducing dual process theories in general and CEST as one of the theories in this category.
5. In line with the previous comment, in the second paragraph of the subsection with the title: “sub-sections of Theoretical foundations of rationality and experientiality”, the authors mention that:
   “Unlike other dual processing models, CEST is presented as a personality theory proposing the two thinking styles as specific variables not fully predicted by classical personality trait topologies (e.g. Big Five explained only 37% of the variance of rationality and 11% of experientiality; Epstein, 2003; Pacini & Epstein, 1999).”

   I don’t know if we have any dual process theories that assume that thinking styles are similar to or predictable by personality traits because thinking style are different concepts compared to personality traits. Please explain more clearly and provide more references. This part and the previous paragraph need to be better explained and more references are required.
6. The materials and methods section should be structured based on the journal guideline.
7. The required sample size should be justified with relevant references.
8. Several headings are repeated in Analytic strategy and results section which create confusion for the reader. Combine these two sections by removing the Analytic strategy part and explain the methods used for conducting different analyses in the relevant parts in the results section.

9. Results section needs an introductory paragraph to explain the logic and the structure of the analyses that follows.
10. Results are not presented in a structured and systematic manner. A more structured and complete results section is required. It is not clear how the subsections are presented. Also, it is not clear why validity is divided into these types of validity. Please refer to other studies and use them in order for conducting proper statistical analyses and presenting your results in a more structured way. Please refer to the journal guidelines for presenting the results in a more structured way.
11. Present the two studies, including the measurements and results in two separate sections. This makes the paper easier to read and understand.
12. Provide references along with more explanations about the methods used to address different types of validity. For example, why correlation and regression tests are used for examining discriminant validity.
13. Comparing females and males scores is not justified. Why the comparison tests are conducted in Table 5. What the results can tell? No rational and no references are provided for the reason that women have higher scores on experientiality compared to men. I think this part is better to be excluded from the paper.
14. Discussion and conclusion sections need to be restructured. We do not generally present the results based on statistical analyses conducted in the study. Revise this section by considering the following questions. What are the major findings based on the results of this study? What are the main theoretical and practical implications? What are the limitations that can be addressed in future studies?
15. The open science and data availability statements are misplaced on page 13 which is the middle part of the paper.

16. The fluency of the paper in English language is not enough. It needs to be revised. In several occasions, especially in the discussion section very long sentences are presented in disorganized way.
