# Supplementary material for: The Serbian validation of the Rational-Experiential Inventory-40 and the Rational-Experiential Multimodal Inventory
Source: PLoS One. 2023 Nov 28;18(11):e0294705. doi: 10.1371/journal.pone.0294705 (PMC10684000; doi:10.1371/journal.pone.0294705)
Supplement: Attachment — Submitted filename: Response to Editor R2.docx [file pone.0294705.s015.docx]

**PONE-D-22-02800: The Serbian validation of the Rational-Experiential Inventory-40 and the Rational-Experiential Multimodal Inventory - Response to reviewers**

We would like to thank the editor for the positive evaluation of our manuscript and additional suggestions for improving the text. Our responses are written in blue below.

Editor’s comments:

One aspect that is not entirely clear is the mention of "credible intervals" on line 212, given that the analyses are not Bayesian. Shouldn't this reference be only to "confidence intervals"?

Yes, you are correct, we used a generic formulation which included credible intervals, however only confidence intervals are appropriate for our study. We have removed this part.

Furthermore, on line 416, I would recommend adding the word 'significant' as follows: "The results revealed essentially no SIGNIFICANT gender differences."

Corrected.

Additionally, on line 530, it should read 'No SIGNIFICANT differences were found...'

Corrected.

Regarding line 534, please specify the value you used for 'small effect sizes.'

Added. We used the same value of d = .20 as in Study 1.
